# Chest Compression Superimposed with Sustained Inflation or 3:1 Compression/Ventilation Ratio During Neonatal Cardiopulmonary Resuscitation in the Delivery Room: A Systematic Review and Meta-Analysis

**DOI:** 10.3390/children12020230

**Published:** 2025-02-13

**Authors:** Jenny Koo, Anup C. Katheria, Brenda Law, Anne Lee Solevåg, Gerhard Pichler, Georg M. Schmölzer

**Affiliations:** 1 Sharp Mary Birch Hospital for Women and Newborns, Neonatal Research Institute, San Diego, CA 92123, USA; jenny.koo@sharp.com (J.K.); anup.katheria@sharp.com (A.C.K.); 2 Centre for the Studies of Asphyxia and Resuscitation, Neonatal Research Unit, Royal Alexandra Hospital, 10240 Kingsway Avenue NW, Edmonton, AB T5H 3V9, Canada; blaw2@ualberta.ca; 3 Department of Pediatrics, Faculty of Medicine and Dentistry, University of Alberta, Edmonton, AB T6G 2R3, Canada; 4 Department of Neonatal Intensive Care, Division of Pediatric and Adolescent Medicine, Oslo University Hospital Rikshospitalet, 0372 Oslo, Norway; a.l.solevag@medisin.uio.no; 5 Department of Pediatrics, Faculty of Medicine, Medical University Graz, 8036 Graz, Austria; gerhard.pichler@medunigraz.at

**Keywords:** chest compressions, newborn, delivery room, cardiopulmonary resuscitation

## Abstract

**Background:** Current resuscitation guidelines recommend a 3:1 chest compression/ventilation (C:V) ratio, which is associated with high morbidity and mortality. An alternative might be continuous chest compression superimposed with high distending pressure or sustained inflation (CC + SI). **Objective:** To compare CC + SI with 3:1 C:V during neonatal cardiopulmonary resuscitation (CPR). **Methods:** MEDLINE (through PubMed), Google Scholar, EMBASE, and Clinical Trials.gov through June 2024. Randomized controlled trials comparing CC + SI with 3:1 C:V during neonatal CPR in the delivery room were included. Data Analysis included Risk of bias was assessed using the Covidence collaboration tool, and results were pooled into a meta-analysis using a fixed effects model. Main **outcomes were** In-hospital mortality (primary). Time to return of spontaneous circulation (ROSC) and air leak (secondary). **Results:** Two studies were included. The pooled data suggests no difference in infant mortality between CC + SI versus 3:1 C:V during neonatal CPR (RR 0.64, 95% CI 0.21,1.7, *p* = 0.33, I^2^ = 63%). The use of CC + SI during neonatal CPR could result in 182 fewer per 1000 (from 351 fewer to 311 more) infant deaths. The pooled data suggested a significant reduction in time to ROSC with CC + SI versus 3:1 C:V during neonatal CPR (mean difference 115 s (from 184.75 to 45.36 s), *p* = 0.001, I^2^ = 26%). Air leak was not different between groups. **Conclusions:** While in-hospital mortality and air leak were not different between groups, time to ROSC was significantly reduced. A large clinical trial is warranted to assess if CC + SI improves outcomes.

## 1. Introduction

Approximately 0.1% of term infants and up to 15% of preterm infants (equating to 2–3 million globally) require chest compressions (CC) at birth in the delivery room [1,2]. Among infants who receive CC, there is a high risk of mortality (41%) and neurological complications, including hypoxic–ischemic encephalopathy and seizures (57%) [3,4,5]. The current guidelines from the International Liaison Committee on Resuscitation (ILCOR) recommend a 3:1 compression-to-ventilation (C:V) ratio during CC, which involves 90 compressions and 30 ventilations per minute, with a pause after every third compression to deliver a ventilation [1,2]. These recommendations are based on findings from adult clinical trials and neonatal animal studies, as randomized controlled trials in newborns are currently unavailable. Urgent clinical trials are needed to identify the most effective neonatal CC approach [1,2].

Providing high-quality CC, to mechanically pump the blood through the body, is critical in achieving return of spontaneous circulation (ROSC). Blood expulsion from the ventricles during CPR occurs by either the cardiac pump theory or the thoracic pump theory [6,7,8,9,10,11]. The “cardiac pump theory” suggests that CC directly expels blood from the heart into circulation with each compression. In contrast, the thoracic pump theory proposes that CC generates a phasic increase in intrathoracic pressure, creating a pressure gradient between arterial and venous compartments that drives forward blood flow [6,7,8,9,10,11]. While performing high-quality CC (applying adequate force and speed) is essential for ROSC, a critical yet often overlooked adverse effect is lung de-recruitment. Each chest compression forces air out of the lungs (forced exhalation), leading to progressive lung de-recruitment [12,13]. For instance, in an adult pig undergoing 7 min of CC-only CPR (without ventilation), lung computed tomography revealed an increase in atelectasis from 25% pre-CPR to 75% post-CPR [13], highlighting significant lung de-recruitment during CC-only CPR. Similarly, in neonatal piglets, a tidal volume loss of up to 4.5 mL/kg per 3:1 C:V cycle was observed, further demonstrating the impact of CC on lung function [12].

An animal study using asphyxia-induced cardiac arrest in neonatal piglets demonstrated that combining chest compressions (CC) with ventilations significantly improves return of spontaneous circulation (ROSC) and neurological outcomes at 24 h compared to ventilations or CC alone [14]. This underscores the critical importance of ventilation in neonatal resuscitation. Studies comparing different compression-to-ventilation (C:V) ratios in asphyxiated neonatal piglets with cardiac arrest showed no significant differences in time to ROSC: 150 s versus 148 s for 3:1 versus 9:3 C:V [15]; 150 s versus 195 s for 3:1 versus 15:2 C:V [16]; and 127 s, 96 s, and 119 s for 2:1, 3:1, and 4:1 C:V ratios, respectively [17]. These findings suggest that varying the C:V ratio does not improve outcomes, likely due to interruptions in CC [18] and lung de-recruitment [12].

Chest compressions combined with sustained inflation (CC + SI) involves delivering a constant high distending pressure (sustained inflation, or SI) during continuous CC. During compressions, air is expelled from the lungs, and during chest recoil, the high distending pressure facilitates gas flow back into the lungs. Animal studies have shown that CC + SI significantly improved hemodynamics (mean arterial pressure: 51 vs. 31 mmHg, *p* < 0.05; pulmonary arterial pressure: 41 vs. 31 mmHg, *p* < 0.05), mean minute ventilation (936 vs. 623 mL/kg, *p* < 0.05), and survival (7/8 vs. 3/8, *p* = 0.038) [19,20,21]. Two porcine studies demonstrated faster median time to ROSC [38 vs. 143 s, *p* = 0.0008, CC + SI vs. 3:1 C:V [19]; 34 vs. 210 s, *p* = 0.048 CC + SI vs. 3:1 C:V [22]]. The advantages of this technique are as follows: (1) sustained inflation raises intrathoracic pressure, significantly enhancing carotid blood flow during CPR without obstructing venous return [6,7] and (2) during each CC cycle, air is expelled during compression and flows back into the lungs during recoil, maintaining ventilation [19,20,21,22,23,24,25]. Unlike the 3:1 C:V method, which results in cumulative tidal volume loss of up to 4.5 mL/kg per cycle, CC + SI achieves a tidal volume gain of up to 2.3 mL/kg per cycle [12].

We performed a meta-analysis and systematic review of clinical trials comparing CC + SI and 3:1 C:V during neonatal CPR. We hypothesized that in newborn infants CC + SI compared to 3:1 C:V will reduce in-hospital mortality and have a faster time to ROSC.

## 2. Methods

This review was conducted with the standard methods of the Cochrane Handbook for Systematic Reviews of Interventions version 5.3. Reporting was in accordance with the Preferred Reporting Items for Systematic Reviews and Meta-Analyses (PRISMA) checklist, which is attached as a supplement. The review has been registered with the International Prospective Register of Systematic Reviews (PROSPERO CRD42022372919). No review protocol was prepared and there were no amendments to the protocol.

We searched the following electronic databases: MEDLINE (through PubMed), Google Scholar, EMBASE, and Clinical Trials.gov using a predefined algorithm (see Appendix A), with the included search terms “infant”, “newborn”, “resuscitation”, “chest compression”. We also performed a manual search of references in articles identified by our search strategy (Appendix A). No language or publication period restrictions were applied. Studies conducted in older children, adults, in settings outside the delivery room or animal studies were excluded. We also excluded studies performed in a simulation setting or using or manikins.

### 2.1. Study Selection

Authors independently assessed the title and abstract of studies for eligibility and authors of a randomized trial examining chest compression were excluded from the eligibility screening. Then, full texts were retrieved and were included based on the eligibility criteria. Any disagreement was resolved through discussion. Only randomized controlled trials comparing CC + SI with 3:1 C:V in the delivery room and reporting mortality were included. Outcomes were chosen according to the consensus outcome rating for international neonatal resuscitation guidelines [26]. Our primary outcome was neonatal mortality in the first 28 days and mortality before discharge. Secondary outcomes included duration of chest compression, time to ROSC, administration of epinephrine, administration of volume expanders, administration of red blood cells, stage of hypoxic–ischemic encephalopathy (Sarnat) [27], lengths of hospital admission, brain injury reported by ultrasound or magnet resonance imaging (including intraventricular hemorrhage [28], periventricular leukomalacia, or hypoxia), intervention in the delivery room (e.g., intubation, mask ventilation, laryngeal mask), lengths of mechanical ventilation in the neonatal intensive care unit, use of inotropes, rate of seizures, endotracheal intubation in the neonatal intensive care unit during hospitalization, air leaks (pneumothorax, pneumomediastinum, pneumopericardium, pulmonary interstitial emphysema), and neurodevelopment follow-up at 18–24 months of age. The review team resolved any discrepancies regarding inclusion through consensus.

### 2.2. Data Extraction

Data extraction was conducted using a standardized collection form that captured details on study design, methods, patient characteristics, interventions, and outcomes. Information regarding randomization methods, allocation concealment, blinding, and adherence to intention-to-treat analysis was also recorded. The Covidence collaboration tool was used to facilitate data extraction. Two investigators (JK and AK) independently extracted the data, with any discrepancies resolved through consultation with another member of the review team.

### 2.3. Assessment of Methodological Quality

The methodological quality and risk of bias of the included trials were evaluated using the risk of bias version 2 tool within Covidence. The assessment covered key domains such as sequence generation, allocation concealment, blinding of participants and personnel, blinding of outcome assessors, incomplete outcome data, selective outcome reporting, and other potential sources of bias. Additionally, two authors (JK and AK) independently assessed the certainty of evidence (confidence in the effect estimates) for each outcome using the Grading of Recommendations Assessment, Development, and Evaluation (GRADE) framework. This included calculating the optimal information size to evaluate imprecision, utilizing the GRADEpro Guideline Development Tool (McMaster University, Hamilton, ON, Canada).

### 2.4. Statistical Analysis

Covidence, GRADEpro, and Review Manager software 5.3 were used to abstract, summarize, and analyze the data, respectively. The principal summary measures were the risk ratio (RR) for dichotomous outcomes and weighted mean difference for continuous outcomes. For each trial, we retrieved or calculated the crude RR estimates and corresponding 95% confidence intervals for the assessed outcomes. Heterogeneity was assessed using an χ^2^ test and the I^2^ statistic. We used random effect models to summarize RR estimates. Where the pooled estimates of RR were statistically significant, we calculated the numbers needed to treat (NNT) for this outcome. All *p*-values are two tailed.

## 3. Results

**Figure 1:**
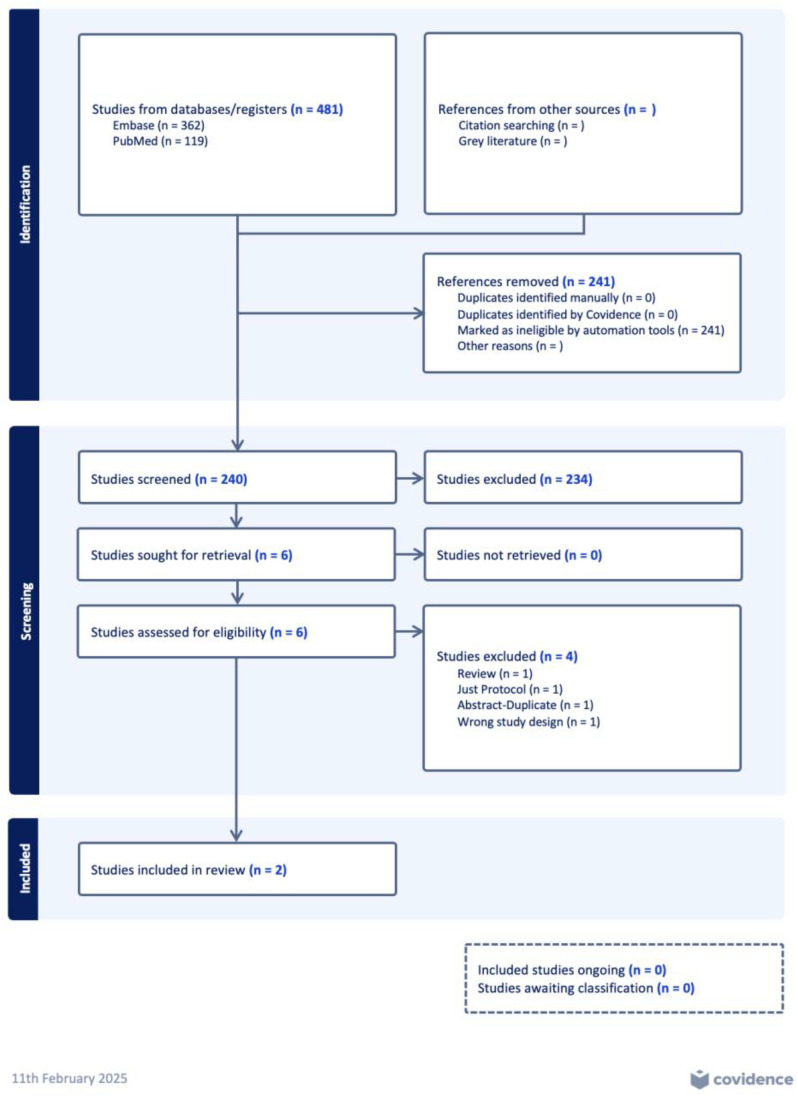
PRISMA flowchart for the selection of eligible studies from Covidence collaboration tool.

Our search obtained 481 records, with a total of 241 records being removed by the automated Covidence tool. From the 240 remaining, 234 records were removed after titles and abstracts screening and six records were screened for their full texts. Four studies were removed due to wrong study design (*n* = 1), a review (*n* = 1), protocol (*n* = 1), and abstract (*n* = 1), respectively. Consequently, two RCTs were included in this review [29,30]. The PRISMA flow diagram is presented in Figure 1 and the GRADE Assessment of Evidence Table for key pre-specified outcomes is shown in Table 1. No ongoing study was found, however, a trial registration (Sustained Inflation and Chest Compression vs. 3:1 C:V Ratio in Asphyxiated Newborns (SUR1VE-2), NCT06577818) was found on Clinical Trials. The Cohen’s Kappa coefficient for title and abstract screening was 0.7.

### 3.1. Characteristics of Included Studies

One RCT enrolled in Canada only and one RCT enrolled in Canada and Austria were both included in this review. A total of 34 newborn infants requiring CC in the delivery room were included in this review [29,30]. Both studies compared CC + SI with 3:1 C:V [29,30]. The Schmölzer et al. trial only included infants 23^+0^ to 32^+6^ weeks [mean (SD) birthweight and gestation 707 (208) g and 24.6 (1.3) weeks with CC + SI and 808 (192) g and 25.6 (2.3) weeks with 3:1 C:V] and was a single-center trial with individual randomization [29]. The SURV1VE-trial included >28 weeks’ gestation infants [median (IQR) birthweight and gestation 2100 (1720–3000) g and 35.4 (31.9–39.1) weeks with CC + SI and 2835 (1270–3700) g and 35.6 (30–40.3) weeks with 3:1 C:V], was a multi-center trial, and used cluster randomization [30]. Characteristics of the included studies are shown in Table 2. The SURV1VE-trial [30] was reported as per the Neonatal UTSTEIN template [31] while the trial by Schmölzer et al. [29] was published prior to the Neonatal UTSTEIN template publication.

### 3.2. Quality of Individual Studies

**Figure 2:**
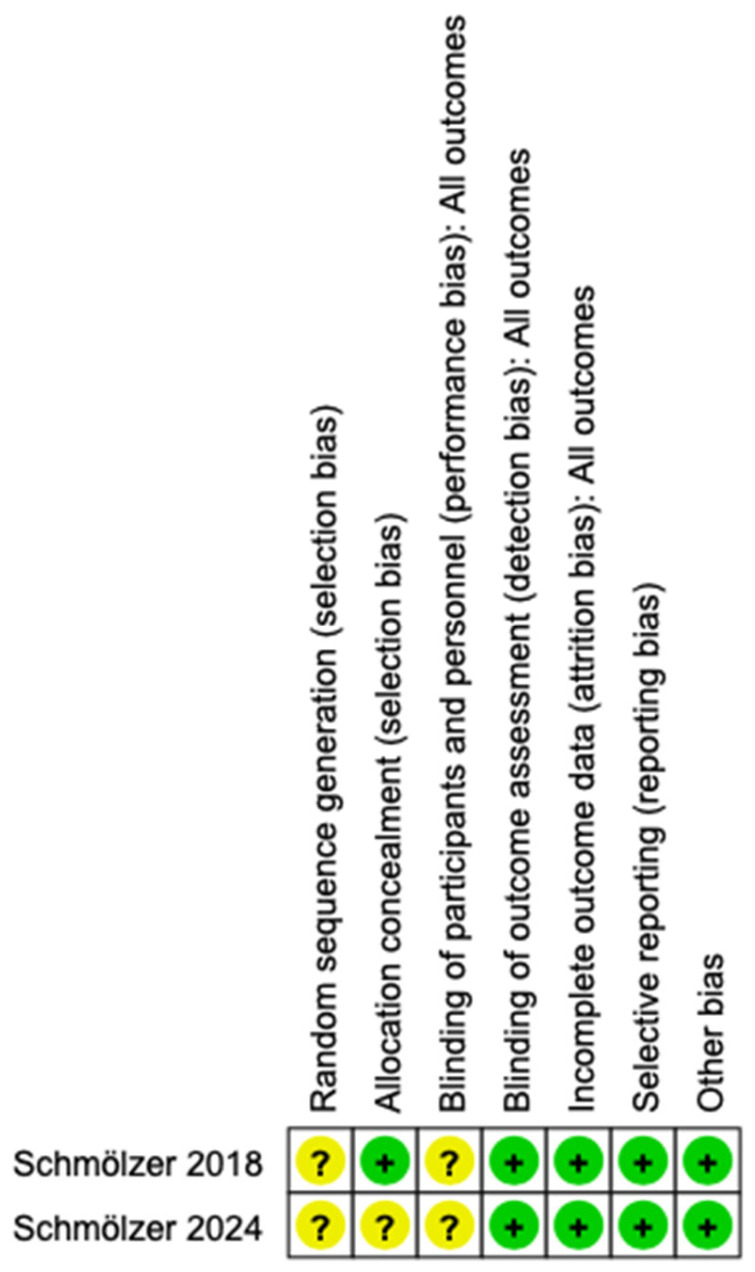
Assessment of risk of bias in the included studies [29,30].

Assessment of potential sources of bias are presented in Figure 2. Risk of bias of the included studies was assessed using the Cochrane collaboration tool. Both studies reported the unclear risk of allocation concealment [29,30], none of the studies reported blinding of participants or personnel but reported blinding of the outcome assessors for the interventions. For incomplete outcome reporting and selective outcome reporting, both studies were at a low risk of bias [29,30]. Other sources of risk of bias were not identified in the studies included.

### 3.3. Primary Outcome

#### Neonatal Mortality in the First 28 Days and Mortality Before Discharge

**Figure 3:**
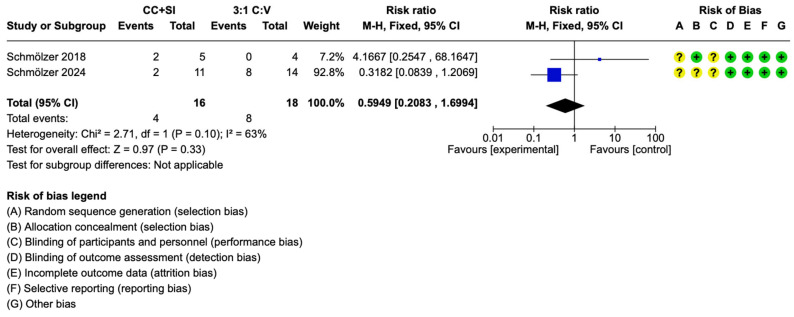
In-hospital mortality [29,30].

Both included studies reported infant mortality [29,30]. The pooled data suggests no difference in infant mortality between CC + SI versus 3:1 C:V during neonatal CPR (RR (95% CI) 0.64 (0.21,1.7), *p* = 0.33, I^2^ = 63%) (Figure 3). The use of CC + SI during neonatal CPR could result in 182 fewer per 1000 (from 351 fewer to 311 more) infant deaths (Table 1).

### 3.4. Secondary Outcomes

**Figure 4:**
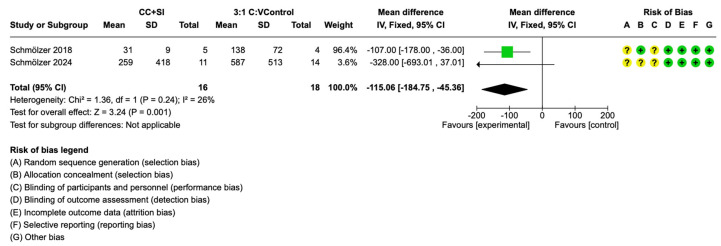
Time to return of spontaneous circulation (ROSC) [29,30].

For the majority of secondary outcomes no data were identified. Secondary outcomes including air leaks, intraventricular hemorrhage all grades, and intraventricular hemorrhage Papile Grade III or IV [28] are presented in Table 1 and Figure 4.

While duration of chest compression was not reported, the time to ROSC was reported in both trials [29,30]. The pooled data suggests a significant reduction in time to ROSC with CC + SI versus 3:1 C:V during neonatal CPR (mean difference (95% CI) 115 (184.75–45.36) s, *p* = 0.001, I^2^ = 26%) (Figure 4)). The use of CC + SI during neonatal CPR could result in a faster time to ROSC by 115 s (from 184 to 45 s) (Table 1).

## 4. Discussion

Neonatal resuscitation guidelines are based on data from adult or pediatric trials and neonatal animal data as there is a lack of neonatal human data. Indeed, the current consensus and treatment recommendations state that clinical trials are urgently needed to examine the optimal neonatal CPR approach [1,2]. This systematic review and meta-analysis identified two small trials comparing CC + SI and 3:1 C:V with a total of 16 infants with CC + SI and 18 infants with 3:1 C:V [29,30,32]. The results can be summarized as follows: (i) there was no difference in the primary outcome of in-hospital mortality between CC + SI vs. 3:1 C:V (Figure 3); (ii) time to ROSC was reduced by a mean of 115 s with CC + SI vs. 3:1 C:V, which was statistically significant (Figure 4); (iii) there was no difference in air leaks between both CC approaches.

Mortality rates of newborns receiving CC are around 40–50%, which significantly increases to 82% in newborns who receive CC and have no signs of life at 10 min after birth [3,4,5]. While one neonatal animal study comparing CC + SI vs. 3:1 C:V reported that significantly more piglets survived to 4 h after resuscitation with CC + SI than in the 3:1 group (seven out of eight [87.5%] versus three out of eight [37.5%], respectively; *p* = 0.038) [19], no other animal study reported differences in mortality [22,24,25,33,34]. The initial pilot trial reported a mortality of two out of five with CC + SI vs. zero out of four with 3:1 C:V (*p* = 0.114) [29]. In the SURV1VE-trial, mortality was 2/11 (18%) with CC + SI vs. 8/14 (57%) with 3:1 C:V, *p* = 0.10 (Fisher’s exact test) and OR (95% CI) 0.17 (0.03 to 1.07) [30]. Similarly, in the current meta-analysis mortality was not different with a pooled RR (95% CI) 0.64 (0.21,1.7)) (Table 1, Figure 3).

The animal study compared CC + SI with a CC rate of 120/min vs. 3:1 C:V and reported a significantly reduced mean (SD) time to ROSC with CC + SI vs. 3:1 C:V (32 (11) s vs. 205 (113) s, respectively) [19]. Further animal studies randomized to CC + SI with CC rates of 90/min vs. CC + SI with CC rates 120/min and reported no difference in time of ROSC with both CC + SI groups [34 (28–156) s vs. 99 (31–255) s, (*p* = 0.29)], and significantly longer time to ROSC with 3:1 C:V (control group) [210 (72–300) s] (*p* = 0.05) [22,25].

The pilot trial in this meta-analysis reported a significantly shorter mean (SD) time to ROSC with CC + SI of 31 (9) s vs. 138 (72) s with 3:1 C:V (*p* = 0.011) [29]. The recently completed SURV1VE-trial compared CC + SI (*n* = 11) with 3:1 C:V (*n* = 14) and reported a median (IQR) time to ROSC of 90 (60–270) s with CC + SI vs. 615 (174–780) s with 3:1 C:V (*p* = 0.0502 (log rank), *p* = 0.16 (cox proportional hazards regression)) [30]. Once the data were pooled in the meta-analysis a significant reduction in time to ROSC with CC + SI vs. 3:1 C:V (mean difference 115 s, 95% CI 184.75–45.36 s, *p* = 0.001, I^2^ = 26%) (Table 1, Figure 4) can be observed. While the pooled analysis indicates a shorter duration to ROSC, the number of infants in the trials is very low and therefore further trials are needed before this can be translated into clinical practice.

CPR methodology must balance providing adequate respiratory and cardiovascular support to achieve ROSC, while limiting iatrogenic injury [21]. Delivery room studies of preterm infants have demonstrated that receiving positive pressure ventilation is associated with increased inflammatory markers [35,36], higher rates of intraventricular hemorrhage [37,38], and increased mortality [39]. However, there are limited data from during neonatal CPR. Neonatal animal CPR studies comparing different C:V ratios or continuous chest compression with asynchronized ventilation with CC + SI did not observe any difference in brain or lung inflammation or oxidative injury [17,22,40,41]. Furthermore, there was no difference in rates of intraventricular hemorrhages in the current meta-analysis between groups (Table 1). Sustained inflation as initial respiratory support within the SAIL trial reported a higher rate of pneumothorax (6% vs. 1% in the control groups, *p* = 0.06) [39]; however, neither neonatal animal CPR studies, nor both clinical trials, and the current meta-analysis reported higher rates of air leaks (Table 1) [19,21,22,24,25,29,32].

The SURV1VE-trial also reported challenges due to the COVID-19 pandemic, challenges to obtain trial insurance, and institutional review boards of different sites communicating that they would like to see more human data before approving the trial at their site [21,30]. These obstacles need to be overcome if a further trial can be conducted. Furthermore, both trials were only conducted in high-resource and high-income settings. To be translatable, a future trial comparing CC + SI with 3:1 C:V is currently planned (NCT06577818) and aims to recruit in high-, middle-, and low-resource settings as well as high-, middle-, and low-income countries as well as having parent representation.

### 4.1. Limitations

Our meta-analysis may have been affected by small sample sizes, no blinding of clinical personnel, and no data on long-term neurodevelopmental outcomes. Furthermore, one trial included infants 23^+0^–32^+6^ weeks’ gestation and the other trial ≥28 weeks’ gestation, which might have influenced the results. In addition, both trials originate from one center and the same group of investigators, which limits the potential generalizability of any findings.

### 4.2. Clinical Applications

Our results might have implications for the delivery room in settings with a T-Piece device as CC + SI reduced the time to ROSC. Paramedics, emergency room personnel, or resource-limited settings might not have a T-Piece and therefore could not use CC + SI.

## 5. Conclusions

There was no difference in in-hospital mortality in newborns receiving CC + SI or 3:1 C:V during cardiopulmonary resuscitation. CC + SI significantly reduced the time to ROSC compared to 3:1 C:V. There was no difference in air leaks or intraventricular hemorrhage between both interventions. A large clinical trial to compare CC + SI with 3:1 C:V during neonatal chest compression in the delivery room is warranted.

## Figures and Tables

**Table 1:** GRADE Quality of Evidence Assessment for primary and pre-specified secondary outcomes.

Certainty Assessment							No. of Patients		Effect		Certainty	Importance
No. of Studies	Study Design	Risk of Bias	Inconsistency	Indirectness	Imprecision	Other Considerations	[Intervention]	[Comparison]	Relative (95% CI)	Absolute (95% CI)		
Neonatal Mortality in the first 28 days or before discharge												
2	randomized trials	not serious	not serious	not serious	serious		4/16 (25.0%)	8/18 (44.4%)	RR 0.59 (0.21 to 1.70)	182 fewer per 1000 (from 351 fewer to 311 more)	-	Critical
Time to ROSC												
2	randomized trials	not serious	not serious	not serious	serious		16	18	-	MD 115.06 s lower (184.75 lower to 45.36 lower)	-	Not reported
Air leak												
2	randomized trials	not serious	not serious	not serious	serious		1/16 (6.3%)	2/18 (11.1%)	RR 0.64 (0.07 to 6.14)	40 fewer per 1000 (from 103 fewer to 571 more)	-	Important
Intraventricular hemorrhage all grades												
2	randomized trials	not serious	not serious	not serious	serious		2/16 (12.5%)	6/18 (33.3%)	RR 0.74 (0.32 to 1.76)	87 fewer per 1000 (from 227 fewer to 253 more)	-	Critical
Intraventricular hemorrhage Papile Grade III or IV [28]												
2	randomized trials	not serious	not serious	not serious	serious		2/16 (12.5%)	4/18 (22.2%)	RR 1.30 (0.46 to 3.70)	67 more per 1000 (from 120 fewer to 600 more)	-	Important

CI: Confidence interval; MD: mean difference; RR: risk ratio.

**Table 2:** Characteristics of the included studies.

Author, Year	Centers	Randomization	Inclusion Criteria	Exclusion Criteria	Intervention	Control	Primary Outcome
Schmölzer, (2018) [29]	Single-center (Edmonton, Canada)	Individual randomization	23^+0^ to 32^+6^ weeks’ gestation	Congenital abnormalities or if parents did not provide consent	CC + SI	3:1 C:V	Time to ROSC
Schmölzer, (2024) [30]	Multi-center (*n* = 4) (Canada and Austria)	Cluster randomization	>28 weeks’ gestation	Congenital abnormalities or if parents did not provide consent	CC + SI	3:1 C:V	Time to ROSC

CC + SI = Chest compression superimposed with sustained inflation, 3:1 C:V = 3:1 compression/ventilation ratio, ROSC = return of spontaneous circulation.

## Abbreviations

CC	chest compression
CPR	cardiopulmonary resuscitation
ILCOR	International Liaison Committee on Resuscitation
C:V	compression to ventilation ratio
ROSC	return of spontaneous circulation
CC + SI	chest compression superimposed with sustained inflation
IQR	Interquartile range
RR	Relative Risk
CI	Confidence interval

## Appendix A. Search Strategy

Pubmed:

((((((Heart Massage[MeSH Terms]) OR heart massage*[Title/Abstract]) OR

cardiac massage*[Title/Abstract]) OR compression*[Title/Abstract])) AND ((“Respiration, Artificial”[Mesh:NoExp]) OR ventilation*[Title/Abstract])))) AND ((ratio

[Title/Abstract]) OR ratios[Title/Abstract]))) NOT ((“letter”[pt] OR “comment”[pt] OR “editorial”[pt] or Case Reports[ptyp])) AND (“Respiratory Distress Syndrome,

Newborn”[Mesh] OR “Bronchopulmonary Dysplasia”[Mesh] OR “Infant, Newborn”[Mesh] OR “Delivery Rooms”[Mesh] OR “Gestational Age”[Mesh] OR

“Premature Birth”[Mesh] OR “Infant, Premature, Diseases”[Mesh:NoExp] OR “Term Birth”[Mesh] OR “Live Birth”[Mesh] OR “Neonatal Nursing”[Mesh] OR

“Neonatal Screening”[Mesh] OR “Intensive Care, Neonatal”[Mesh] OR “Intensive Care Units, Neonatal”[Mesh] OR “ Newborn”[Mesh] OR “Transient

Tachypnea of the Newborn”[Mesh] OR “Persistent Fetal Circulation Syndrome”[Mesh] or newborn[TIAB] or neonatal[TIAB] or neonate[TIAB] or neonates[TIAB]

OR “Low Birth Weight “[TIAB] or “Small for Gestational Age”[TIAB] or prematur*[TIAB] or preterm[TIAB] OR “Birth Injuries”[Mesh] OR “Birthing Centers”[Mesh]

OR Postmature[TIAB] OR infants[TIAB] OR infant[TIAB] OR birth[TIAB])

Embase:

Exp Heart Massage OR Exp cardiac OR exp compression AND

Exp Respiration, Artificial OR exp ventilation AND

exp neonatal respiratory distress syndrome

OR exp newborn hypoxia OR exp prematurity OR exp newborn apnea OR exp newborn disease

OR exp neonatal stress OR exp lung dysplasia OR exp newborn OR exp low birth weight OR exp newborn screening OR exp newborn monitoring OR exp newborn care OR exp newborn period

OR exp birth weight OR exp newborn morbidity OR exp live birth OR exp newborn death

OR exp newborn mortality OR exp delivery room OR exp newborn OR exp low birth weight

OR exp small for gestational age OR exp prematur* OR exp preterm OR exp postmature OR exp macrosomia

Google Scholar

Heart Massage OR heart massage* OR cardiac massage* OR compression AND Respiration, Artificial OR ventilation AND ratio AND Respiratory Distress Syndrome OR Bronchopulmonary Dysplasia Infant OR Newborn OR Delivery Rooms OR Gestational Age OR Premature Birth Infant OR Premature OR Diseases OR Term Birth OR Live Birth OR Neonatal Nursing OR

Neonatal Screening OR Neonatal Intensive Care OR Neonatal Intensive Care Units OR Transient

Tachypnea of the Newborn OR Persistent Fetal Circulation Syndrome OR neonatal OR neonate OR neonates OR Low Birth Weight OR Small for Gestational Age OR prematur* OR preterm OR Birth Injuries OR Birthing Centers OR Postmature OR infants OR infant OR birth
